# Long-term peritoneal dialysis and encapsulating peritoneal sclerosis in children

**DOI:** 10.1007/s00467-008-0982-z

**Published:** 2010-01-01

**Authors:** Masataka Honda, Bradley A. Warady

**Affiliations:** 1 Department of Pediatric Nephrology, Tokyo Metropolitan Children’s Hospital, Umezono 1–3–1, Kiyoseshi, Tokyo, 204–8567 Japan; 2 Pediatric Nephrology, The Children’s Mercy Hospital, Kansas City, Missouri USA

**Keywords:** Children, EPS, Japan, Long-term PD, Peritoneal dialysis, Peritoneal sclerosis

## Abstract

Encapsulating peritoneal sclerosis (EPS) is the most serious complication of long-term peritoneal dialysis (PD), with a mortality rate that exceeds 30%. There have been many reports of the incidence of EPS being strongly correlated to the duration of PD. Patients on PD for longer than 5 years, and especially those receiving this treatment for more than 8 years, should undergo careful and repeated surveillance for risk factors associated with the development of EPS. The development of ultrafiltration failure, a high dialysate/plasma creatinine ratio, as determined by the peritoneal equilibration test, peritoneal calcification, a persistently elevated C-reactive protein level, and severe peritonitis in patients on PD for longer than 8 years are signals that should prompt the clinician to consider terminating PD as a possible means of preventing the development of EPS. The impact of the newer, biocompatible PD solutions on the incidence of EPS has not yet been determined.

## Long-term PD and EPS

Encapsulating peritoneal sclerosis (EPS) is the most serious complication of peritoneal dialysis (PD), with a mortality rate that exceeds 30% [1]. There have been many reports of EPS being strongly related to the longevity of PD [2–9]. This association has primarily been reported in Japanese pediatric and adult patients as there are many long-term PD patients in Japan due to the unavailability of deceased donor transplantation.

In this review of EPS, we address the following issues: the relationship between EPS and long-term PD, the criteria for termination of PD in patients at risk for EPS, and the recent prevalence of long-term PD in Japanese children and it’s impact on the development of EPS.

## Definition and clinical features of EPS

Current definitions of EPS are practical and clinically relevant. The multiplicity of suspected etiologies and confusion over the pathology of EPS is reflected in the variety of descriptive terms that exist, such as peritoneal fibrosis, calcific peritonitis, abdominal cocoon, and sclerosing peritonitis. The more accurate term is, in fact, EPS, as it best describes the morphological changes that occur [1]. Whereas the best literal definition of EPS is based on clinical–pathological criteria, the diagnosis of EPS is most often based on the criteria established by an ad hoc committee of the International Society for Peritoneal Dialysis in which the clinical suspicion of the disorder is confirmed by radiological findings [1].

Encapsulating peritoneal sclerosis is characterized by partial or diffuse bowel obstruction, accompanied by marked sclerotic thickening of the peritoneal membrane. The clinical features vary, but they frequently include abdominal pain, nausea, vomiting, weight loss, low-grade fever, hemorrhagic effluent, ultrafiltration failure, ascites, and resistance to recombinant human erythropoietin [1]. The elements of the radiological findings that are pertinent for confirmation of the diagnosis of EPS include verification of peritoneal thickening and encapsulation, identification of intestinal obstruction, evidence of “cocooning”, and the presence of calcification, as detected by ultrasound and/or computed tomography (CT). The latter technique is preferred by the authors. The cocoon appearance is secondary to the presence of a thick fibrous layer encapsulating the small intestine. Although a precise pathological definition of EPS is not currently available, a common feature appears to be a complete loss of the mesothelium, accompanied by gross interstitial thickening within the peritoneal membrane. This thickened interstitium can be cellular (presumably activated fibroblasts) or acellular (presumably interstitial collagen deposition) in nature [1].

The Pediatric EPS Registry was initiated in Japan in 1996 with the aim of establishing better documentation on the incidence and natural history of the disorder in a high-risk population. Of the 843 patients under 16 years of age who received PD between 1981 and the end of 1999, 17 were diagnosed with EPS; this is an incidence of 2.0%, which is similar to the 0.7–2.8% incidence reported among adults participating in large-scale national studies [3, 9] (Table 1). The mortality associated with EPS is reported to be 35–69%, and most survivors require long-term parenteral nutrition because of their inability to maintain an adequate nutritional state by the enteral route [1–3, 5, 10].

**Table 1:** Surveys of encapsulating peritoneal sclerosis

References	Rigby [5]	Nomoto [4]	Kawanishi [7]	Nakamoto [6]	Kawanishi [8]	Hoshii [9]
EPS cases/PD population	54/7374	124/7343	106/3760	256/11549	48/1958	17/843
Incidence rate	0.7%	1.7%	2.8%	2.2%	2.5%	2.0%
Mean PD duration in EPS patients (months)	52 (8–127)	82.9	87 (4–198)	99.6 (10–168)	114 (36–201)	124 ± 36
Mortality rate	56%	31.5% (1 year)	27% (1 year)	39.1%	22.9% (1 year)	12%^a^
		41.7% (2 years)	42% (2 years)		35.4% (2 years)	
		62% (3 years)				

*EPS* Encapsulating peritoneal sclerosis; *PD* peritoneal dialysis

^a^Data primarily from acute stage of illness

A variety of factors are presumably involved in the development of EPS. Although the entity is not unique to dialysis patients, possible causal factors in those patients who develop EPS in association with PD include the duration of the PD, peritonitis, the acetate dialysis solution, the glucose-based/hypertonic dialysis solution, chlorhexidine, and plasticizers [1]. There is also the possibility that some patients are genetically predisposed to develop EPS when exposed to one or more of the aforementioned etiologic agents, but this has not yet been confirmed [1, 2]. Kawaguchi et al. concluded that prolonged PD duration constitutes the single most significant risk factor for EPS and hypothesize that the most frequent primary cause is the cumulative exposure of the peritoneum to bioincompatible PD solutions characterized by the inclusion of glucose and glucose degeneration products (GDPs) [2]. A history of severe peritonitis and/or non-resolving peritonitis, especially in the long-term PD patient, is also particularly problematic and likely contributory [1].

## The relationship between EPS and long-term PD

The relationship between long-term PD and EPS was reported by Nomoto et al. [4] from Japan in 1996 and by Rigby and Hawley in Australia and New Zealand in 1998 [5]. Subsequent to those publications, many other reports have originated from Japan on the subject, with reference to both pediatric and adult patients [6–9]. In the study by Rigby and Hawley, the overall incidence of EPS increased progressively with the duration of PD, with rates of 1.9, 6.4, 10.8, and 19.4% for patients on peritoneal dialysis for <2, 5, 6, and 8 years, respectively [5]. Kawanishi and Kawaguchi [7] found that the incidence of EPS among patients who had been on PD for more than 60 months was as high as 8.0%, although the overall incidence was 2.8%. More recently, Kawanishi et al. reported an overall incidence of 2.5% from Japan, with incidence rates of 0, 0.7, 2.7, 5.9, 5.8, and 17.2% after 3, 5, 8, 10, 15, and >15 years on continuous PD, respectively (Table 2) [8]

**Table 2:** Incidence and outcome of EPS in relation to time on PD [9]

PD duration (years)	Number of patients	EPS cases (incident rate, %)	Mortality (%)	Recovery (%)
<3	337	0		
3 to <5	554	4 (0.7)	0 (0)	4 (100)
5 to <8	576	12 (2.1)	1 (8.3)	10 (83.3)
8 to <10	239	14 (5.9)	4 (28.6)	6 (42.9)
10 to 15	223	13 (5.8)	8 (61.5)	2 (15.3)
>15	29	5 (17.2)	5 (100)	0 (0)
Total	1958	48 (2.5)	18 (37.5)	22 (45.8)

Values are presented as the number (*n*), with the percentage given in parenthesis

Based on their study of pediatric patients, Hoshii et al. reported that patients who developed EPS had all received PD for longer than 5 years, with a mean PD duration of 10.3 years [9]. The incidence of EPS was 6.6% among all patients on PD for longer than 5 years and 22% among those who had received PD for longer than 10 years. Of the 17 patients (83%) who developed EPS, 14 did so after 1994 because the number of long-term PD patients increased in Japan after that date. These data emphasize the need to pay special attention to long-term PD patients with respect to this disorder.

## Peritonitis and EPS

As mentioned above, the second factor most widely recognized as likely increasing the risk for EPS is peritonitis, particularly if the peritonitis is severe, recurrent, or non-resolving in nature [1, 2]. The combined effect of bioincompatible PD solutions and the superimposition of severe peritoneal inflammation caused by infection has been suggested to be of particular importance (the “two-hit” concept) [11]. However, many long-term PD patients do not develop EPS despite experiencing multiple episodes of peritonitis. Likewise, EPS is not infrequently seen in patients who have never experienced infectious peritonitis. Kawanishi et al. found that only 37 of 50 patients treated for EPS had a history of peritonitis [3].

In Japanese children, the rate of peritonitis has been reported to be 0.43 episodes per patient per year in patients diagnosed with EPS. Although nine of 17 (53%) patients with EPS had infectious peritonitis immediately prior to the development of EPS, the overall peritonitis rate in patients with EPS was not significantly higher than the rate of 0.40 episodes per patient per year seen in all patients registered in the Japanese Pediatric PD registry over the same period of observation [9, 12]. Therefore, whereas peritonitis itself may be not a significant risk factor, the risk may be different when the infection occurs in a patient on long-term (>5 years) PD.

## Prevention of EPS

In some cases, discontinuing PD and eliminating exposure to the PD-related risk factors may prevent the development of EPS. However, EPS has been reported to occur after the patient has been transferred to hemodialysis (HD): in 33 of 48 (69%) adult patients with EPS reported by Kawanishi et al. [3] and in five of 17 (29%) children with EPS reported by Hoshii et al. [9], the EPS occurred after their withdrawal from PD. It was assumed that the development of EPS was probably not an effect of the withdrawal itself and that these patients most likely would have developed EPS even if they had continued on PD. However, it has been hypothesized that leaving the peritoneum dry may exacerbate the pathological mechanisms, resulting in the disorder and, in turn, explain the relationship between the clinical appearance of EPS and the termination of PD.

If one hopes to prevent the development of EPS, potentially by terminating PD in a timely manner, it is important to be able to identify clinical features that may stimulate a change in dialysis modality. Unfortunately, this strategy is complicated by the fact that EPS is characterized by its insidious nature, with presenting symptoms that may be vague and nonlocalizing. In addition, signs that may potentially indicate a pre-EPS state, such as a decline in ultrafiltration (UF) capacity or a change to a high-average or high transport state based on a peritoneal equilibration test (PET) evaluation, are not pathognomonic for EPS [2, 3].

In 1998, the Japanese EPS Study Group did publish recommendations for when PD should be discontinued. The recommendations consisted of UF failure and 6 minor criteria [13]. Minor criteria include bloody dialysate and calcification of the peritoneum, PD duration of longer than 8 years, a persistently positive C-reactive protein level, recurrent peritonitis, and development of a high peritoneal membrane transport state by PET.

In 1996, a protocol for peritoneal biopsy of children on PD was initiated in Japan as another means by which EPS could potentially be prevented [14]. The criteria for biopsy consisted of the following: (1) loss of UF capacity and/or calcification of the peritoneum on abdominal CT in patients receiving PD for longer than 5 years or (2) all patients who had received PD for longer than 8 years. The patients were subsequently divided into two groups based on the histological findings: (1) peritoneal sclerosis was characterized by the absence of mesothelial cells, thickening of the submesothelial connective tissue layers, degenerative collagen fibers, and marked thickening of the walls of the microvasculature with narrowing of the vascular lumen; (2) peritoneal fibrosis was characterized by an increase of collagen fibers, the presence of mesothelial cells, and abundant fibroblasts. If peritoneal sclerosis was diagnosed, PD was discontinued. In addition to the biopsy criteria, termination of PD was considered in patients who had received PD for longer than 8 years and who had experienced infectious peritonitis. Finally, when patients who changed dialysis modality initiated HD, prednisolone (1 mg/kg daily) was prescribed to hopefully minimize any peritoneal inflammatory process, followed by a gradual decrease in dosage over 6 months. The use of this approach has been associated with the absence of EPS in six patients who had received long-term PD (9.3 ± 2.0 years) and whose biopsies showed peritoneal sclerosis [14]. A single patient (PD duration: 8 years) did, however, recently die as a result of EPS that developed soon after an episode of peritonitis; he did not have UF failure nor did he demonstrate peritoneal sclerosis on the protocol biopsy [15].

Kawaguchi et al. published guidelines in which they recommend that an alert should be instituted when patients have received PD for longer than 8 years [2]. It should be emphasized here that PD can be continued for longer than 8 years only if certain conditions can be met:

- stable dialysate/plasma creatinine (D/P Cr) ratio based on the PET;
- no evidence of high peritoneal transport capacity;
- no requirement for frequent use of hypertonic dialysis solutions;
- no continuous increase in serum C-reactive protein level;
- clinical stability with good appetite and no signs of overhydration;
- absence of recurrent peritonitis;
- acceptance of increased risk for complications.

Although these criteria were primarily developed for adults, they are probably applicable to children as well, despite the absence of supportive pediatric data. It is hoped that the future development of a surrogate marker for EPS and the increased use of biocompatible dialysis solutions will further reduce the incidence of EPS.

## The recent prevalence of long-term PD in Japanese children

In Japan, 25% of 807 children less than 16 years of age who had received PD as of 1997 had been treated for more than 5 years [16]. There are no similar data of long-term PD therapy in children from other countries. For example, approximately 80% of children who received PD and who are registered in the dialysis database of the North American Pediatric Renal Trials and Collaborative Studies (NAPRTCS) terminated PD in less than 3 years, primarily to receive a kidney transplant [17]. In Japan, however, the number of children on long-term PD has recently decreased because of the current policy—initiated following the publication of the previously mentioned guidelines in 1998 [13]—of withdrawing patients from PD after a treatment period of 8 years in order to avoid the occurrence of EPS. In a historical context, the number of long-term pediatric PD patients had, up to the initiation of this policy, increased by the end of each year, with 100 patients (33% of total patients) having received PD for more than 5 years and 43 patients (14% of total patients) having been on PD for more than 8 years at the end of 1997. The current policy has resulted in a decrease in the overall number of long-term PD patients and especially in the number of patients on PD for more than 8 years (13 patients, 6%) at the end of 2003, despite the absence of any significant change in the incidence of new patients.

**Fig. 1:**
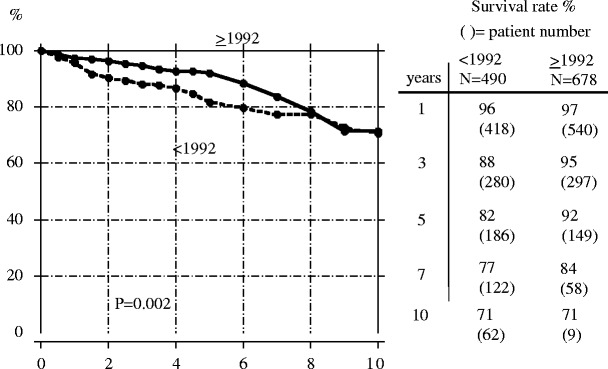
Patient survival rate in children initiating peritoneal dialysis before (*broken line*) and during/after 1992 (*solid line*). Cited from Honda [12]

**Fig. 2:**
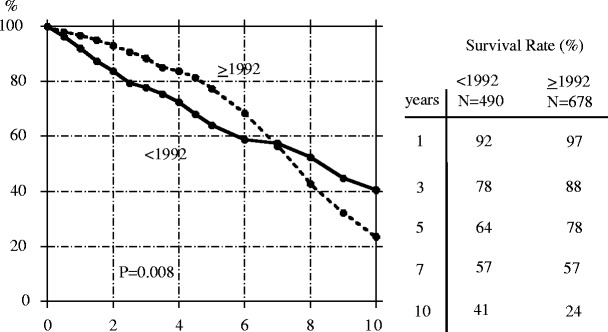
Technique survival rate in children initiating peritoneal dialysis before (*solid line*) and during/after 1992 (*broken line*). Cited from Honda [12]

The patient and technique survival rates of pediatric patients in the Japanese PD registry based on Kaplan–Meier analysis are described in Figs. 1 and 2. Patients who received a kidney transplant or who were transferred to HD were considered lost to observation at the time of transfer in the calculation of patient survival rate. Patients who received a transplant were also counted as lost to observation in the calculation of the technique survival rate. Patients were divided into two groups: patients starting PD in 1981–1991 and those starting PD in 1992–2003. These data demonstrate that the patient survival rate has improved significantly in the most recent era (*p* < 0.002), with a 5-year patient survival rate of 92% in those patients who had initiated PD during or after 1992. These excellent results are similar to the 5-year survival data for the transplant population reported by the United States Renal Data System (USRDS) [18]. In contrast, after 8 years on PD, the survival rate of the more recent cohort decreased to a rate similar to that experienced by those on PD prior to 1992 (Fig. 1).

The technique survival rate for PD has also significantly improved: at 5 years, the technique survival was 78% for patients starting PD during or after 1992 compared to 64% for patients on PD before 1992. However, as was the case with patient survival, no difference in technique survival was seen for the two eras when patients had received PD for more than 8 years (Fig. 2). These results provide evidence that patients on long-term PD (>5 years) are indeed at increased risk for serious complications. In fact, it has been suggested that informed consent be obtained for any patient receiving PD for longer than 8 years because of these issues, even if the patient is clinically well and without risk factors for EPS.

The causes of death and transfer to HD for the Japanese patients who initiated PD after 1991 are shown in Table 3. For those patients who had received PD for more than 5 years, PD was terminated in 44 cases (29%) because the patient underwent transplantation. Transfer to HD occurred in 14 cases in which PD was discontinued secondary to peritonitis and in 13 patients who discontinued PD as a result of UF failure. Death due to peritonitis (two patients) and EPS (one patient) was only seen in long-term PD patients. The more recent absence of EPS as a cause for PD termination is likely the result of a more proactive approach by Japanese physicians to limit long-term PD.

**Table 3:** Outcome and causes of PD termination in children after 1991 [12]

		< 5 years,* n* (%)		≥ 5 years,* n* (%)	
Total		527		151	
Continuance of PD		148 (28)		43 (29)	
Transplantation		244 (46)		44 (29)	
Recovery		14 (3)		1 (1)	
Unknown, lost		37 (10)		8 (6)	
Transfer to HD		50 (10)		40 (27)	
Cause of transfer to HD	Peritonitis	28 (56)		14 (35)	
	UF failure	7 (14)		13 (33)	
	Insufficient dialysis	6 (12)		5 (13)	
	Catheter trouble	3 (6)		0 (0)	
	Others, Unknown	6 (12)		8^a^ (20)	
Death		34 (7)		15 (10)	
Cause of death	Peritonitis	0 (0)		2 (13)	
	Sepsis	1 (3)		1 (7)	
	Pneumonia	4 (12)		1 (7)	
	Cerebro-vascular disorder	2 (6)		2 (13)	
	Heart failure	6 (18)		2 (13)	
	Pulmonary edema	4 (12)		0 (0)	
	Shock, sudden death	4 (12)		0 (0)	
	Others, Unknown	13 (38)		7^b^ (53)	

*HD* Hemodialysis; *UF* ultrafiltration

^a^Long-term PD (three patients), after surgery (two), patient’s request (two), psychological problem (one)

^b^Convulsion (one patient), EPS (one), unknown (two) original disease (two), gastric perforation (one)

## Summary

The performance of long-term PD in Japanese children has been a common practice because of the shortage of deceased donors for transplantation. The results observed in patients who have received PD for up to 5 years have been good. In contrast, the outcome has not been nearly as good for those patients on PD for longer periods of time (e.g. >8 years), with the potential development of EPS a significant concern. Based on current clinical data in children and adults and in the absence of a reliable marker of EPS, strong consideration should be given to electively discontinuing PD in those patients who have received long-term PD (possibly more than 5 years and certainly more than 8 years) and who demonstrate risk factors for EPS, such as UF failure, a high D/P creatinine based on PET assessment, peritoneal calcifications, a persistently elevated C-reactive protein level, and recurrent or severe peritonitis, as a possible means of preventing EPS. Failure to do so is likely to result in a poor patient outcome. Future research will determine whether this scenario can be prevented with the use of the newer, biocompatible PD solutions.

## Questions on long-term PD

(Answers appear following the list of questions)

1. What are the two most common reasons, other than transplantation, for terminating long-term PD in Japanese children who have received PD for > 5 years?
  - Ultrafiltration failure and peritonitis
  - Ultrafiltration failure and cardiovascular disease
  - Cardiovascular disease and peritonitis
  - Fatigue of the patient or caregivers and peritonitis
  - Fatigue of the patient or caregivers and cardiovascular disease
2. Which of the following statements pertaining to EPS is correct?
  - EPS is diagnosed only based on pathological findings
  - The mortality rate of EPS is significantly less than 30%
  - Peritonitis is the most frequent cause for EPS
  - Abdominal symptoms such as bowel obstruction and peritoneal calcifications are important findings in patients with EPS
  - EPS does not occur after the transfer from PD to HD
3. After how many years of PD should we start to pay attention to the possible development of EPS?
  - 3 years
  - 5 years
  - 8 years
  - 10 years
  - 12 years
4. Which of the following findings is not a reason to consider terminating PD as a means of preventing the development of EPS?
  - Persistently positive C-reactive protein
  - Increased D/P creatinine by PET
  - Decreased ultrafiltration capacity
  - Calcification of peritoneal membrane
  - Cardiovascular disease
5. After how many years of PD should informed consent be considered, even in patients who are clinically well, because of the risk of EPS?
  - 3 years
  - 5 years
  - 8 years
  - 10 years
  - 12 years
